# Transformation from polycythemia vera to acute promyelocytic leukemia: Case report and literature review

**DOI:** 10.1097/MD.0000000000030064

**Published:** 2022-08-12

**Authors:** Wen-Wen Li, Xiu-Fang Sui, Shuang Fan, Hong Xu, Cheng-Lei Wang, Fei-Ying Wang, Xiao-Dong Mo

**Affiliations:** a Qingdao Women and Children’s Hospital, Department of Hematology, Qingdao, China; b Peking University People’s Hospital, Peking University Institute of Hematology, National Clinical Research Center for Hematologic Disease, Beijing Key Laboratory of Hematopoietic Stem Cell Transplantation, Beijing, China; c The Affiliated Hospital of Qingdao University, Department of Hematology, Qingdao, China; d Research Unit of Key Technique for Diagnosis and Treatments of Hematologic Malignancies, Chinese Academy of Medical Sciences, Beijing, China.

**Keywords:** acute promyelocytic leukemia, case report, polycythemia vera, transformation

## Abstract

**Introduction:**

Transformation from chronic myeloproliferative neoplasm to acute leukemia is a feature of myeloproliferative neoplasm; however, the rate is not high. Transformation to acute promyelocytic leukemia is rare. Here, we report a case of transformation of polycythemia vera to acute promyelocytic leukemia and describe a process of clonal evolution that has not yet been reported.

**Patient concerns:**

In this case, a 51-year-old woman was diagnosed with polycythemia vera and concomitant JAK2/V617F mutations in July 2019. She underwent intermittent phlebotomy and oral hydroxyurea irregularly. After 2 years, the patient complained of fatigue and poor sleep quality for 2 months.

**Diagnosis:**

Further examination revealed marked hypercellularity and grade 1 bone marrow fibrosis with the PML/RARαV variant (23.85% mutation load), WT1-Exon1 (37.8%), WT1-Exon9 (4.1%), JAK3-Exon7 (49.3%), and RELN-Exon55 (45.8%). According to the World Health Organization classification of tumors of hematopoietic and lymphoid tissues, the patient was ultimately diagnosed with a rare transformation of polycythemia vera to acute promyelocytic leukemia.

**Interventions:**

The patient underwent dual induction therapy with all-trans-retinoic acid and arsenic trioxide.

**Outcomes:**

After 28 days of induction therapy, the patient achieved complete remission, was compliant and the treatment was well tolerated.

**Conclusion:**

Polycythemia vera can transform into acute promyelocytic leukemia; therefore, it is important to review bone aspiration and other tests to perform a comprehensive assessment and monitor the disease status, to detect disease progression and intervene early when it transforms into acute promyelocytic leukemia.

## 1. Introduction

Transformation of chronic myeloproliferative neoplasm (MPN) to acute leukemia is a feature of MPN; however, the rate remains relatively low. This rate varies greatly according to the patient’s clinical and morphological characteristics at the initial diagnosis. Excluding chronic myeloid leukemia, the highest rate is the transformation of primary myelofibrosis, with a 10% to 20% transformation rate at 10 years, followed by polycythemia vera (PV) at 2% to 4% and essential thrombocythemia (ET) at 1%.^[1–6]^

However, transformation to acute promyelocytic leukemia (APL) is rare. Here, we report a case of transformation of PV to APL and describe a process of clonal evolution that has not yet been reported. We also reviewed the literature on the transformation of chronic MPNs into APL.

### 1.1. Case presentation

**Figure 1.**
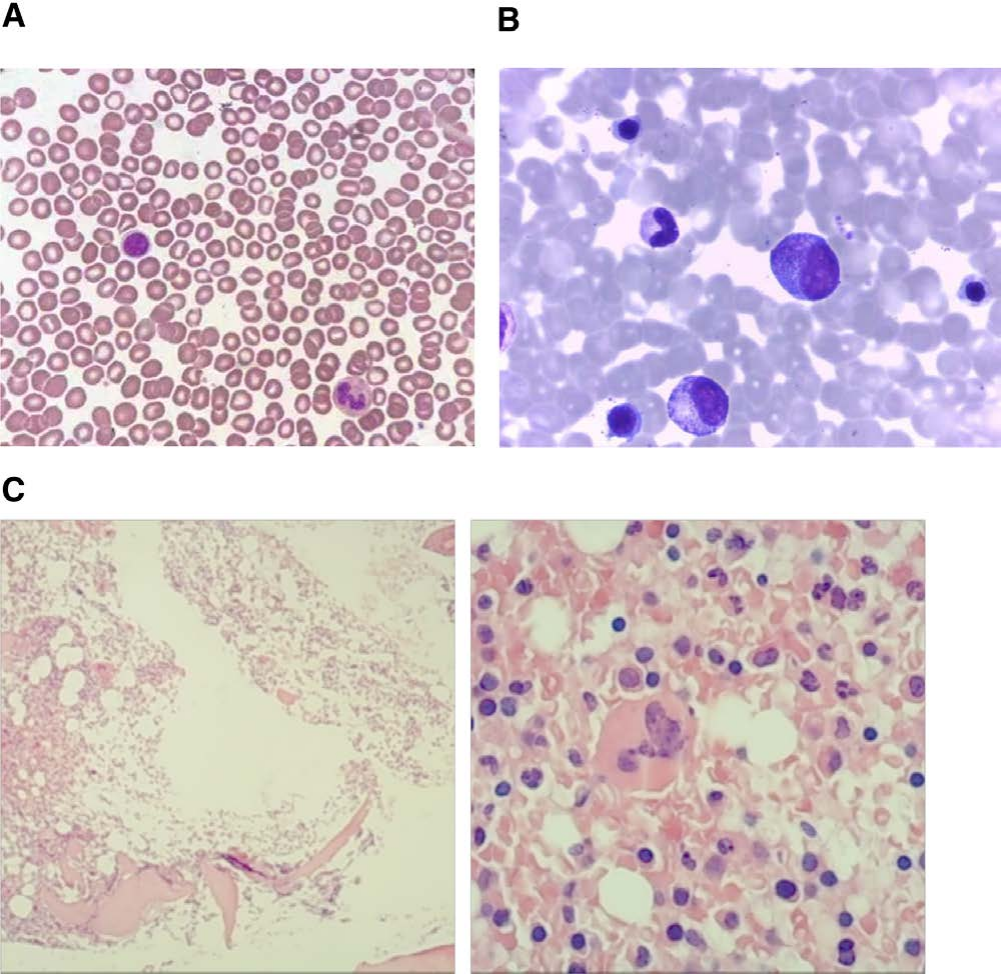
Morphologic features in peripheral blood, bone marrow aspirate and bone marrow biopsy at the time of diagnosis with PV. (A) Morphologic review of the peripheral blood smear revealed that high WBC count, no naive granulocytes and nucleated red blood cells, the red blood cells were normal in size and shape, and more platelets can be seen. (B) The bone marrow aspirate at the time of diagnosis with PV showed hypercellular bone marrow with a G/E ratio of 1.22:1. Erythroid hyperplasia was prominent, mainly in polychromatophilic and late erythroblasts. (C) Bone marrow biopsy at the time of diagnosis with PV revealed prominent hyperplasia (approximately 70%) with grade 1 reticular fibers. G/E = granulocyte/erythroid, PV = polycythemia vera, WBC = white blood cell.

**Figure 2.**
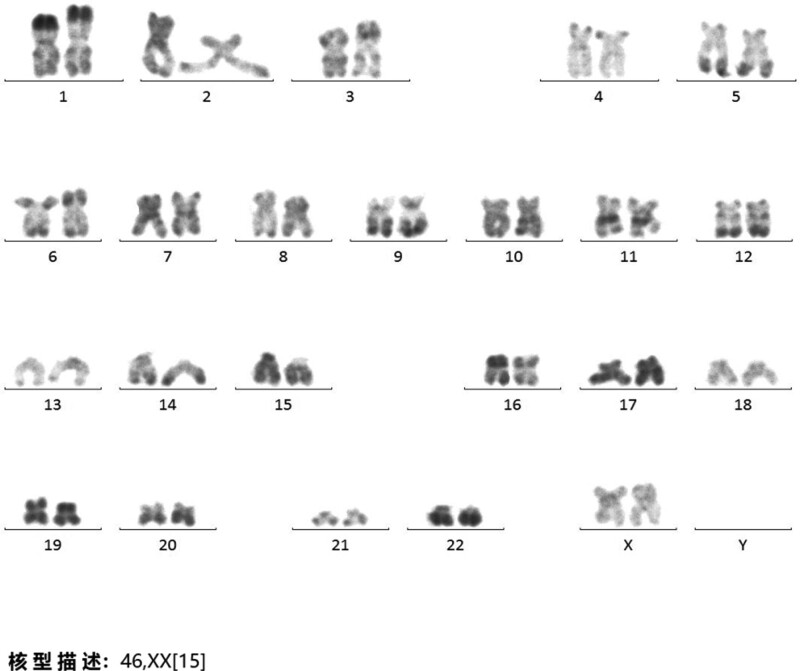
Cytogenetic analysis at the time of diagnosis with PV showed a normal karyotype (46, XX). PV = polycythemia vera.

**Figure 3.**
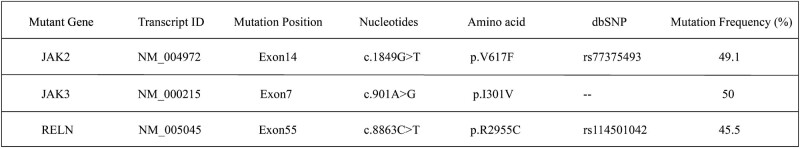
Genetic tests revealed the JAK2-V617F variant (35.18% mutation load), JAK3-Exon7 (50.0%), and RELN-Exon55 (45.5%). dbSNP = The Single Nucleotide Polymorphism Database, ID = Identity document.

A 51-year-old woman had a medical history of cerebral infarction for more than 1 year and hypertension with a maximum blood pressure of 180/120 mmHg. She had pruritus, occasional headache, constipation, fever, redness of the hands and feet, and redness of the face and eyes. In July 2019, complete blood cell count (CBC) results showed: white blood cell (WBC) 7.89 × 10^9^/L, neutrophils 6.47 × 10^9^/L, hemoglobin (Hb) 214 g/L, platelets (PLTs) 548 × 10^9^/L, and mean corpuscular volume 85 fl. Morphologic review of the peripheral blood smear revealed that high WBC count, no naive granulocytes and nucleated red blood cells, the red blood cells were normal in size and shape, and more PLTs can be seen (Fig. 1A). The bone marrow (BM) aspirate showed hypercellular BM with a granulocyte/erythroid ratio of 1.22:1. Erythroid hyperplasia was prominent, mainly in polychromatophilic and late erythroblasts, and cell division was visible. The red blood cells were normal in size and shape. Lymphocytes accounted for 15% of WBC. A total of 21 megakaryocytes were observed throughout the film (Fig. 1B). BM biopsy revealed prominent hyperplasia (approximately 70%) with grade 1 reticular fibers (Fig. 1C). Cytogenetic analysis revealed normal karyotype (46, XX) (Fig. 2). Genetic tests revealed the JAK2-V617F variant (49.1% mutation load), JAK3-Exon7 (50.0%), RELN-Exon55 (45.5%) (Fig. 3). The MPL, CALR, BCR/ABL, and PML/RARα fusion genes were all negative. Erythropoietin levels decreased. According to the 2008 World Health Organization classification of tumors of hematopoietic and lymphoid tissues,^[7]^ the patient was diagnosed with PV. She underwent intermittent phlebotomy and oral hydroxyurea irregularly and reported that Hb levels remained within the normal range.

**Figure 4.**
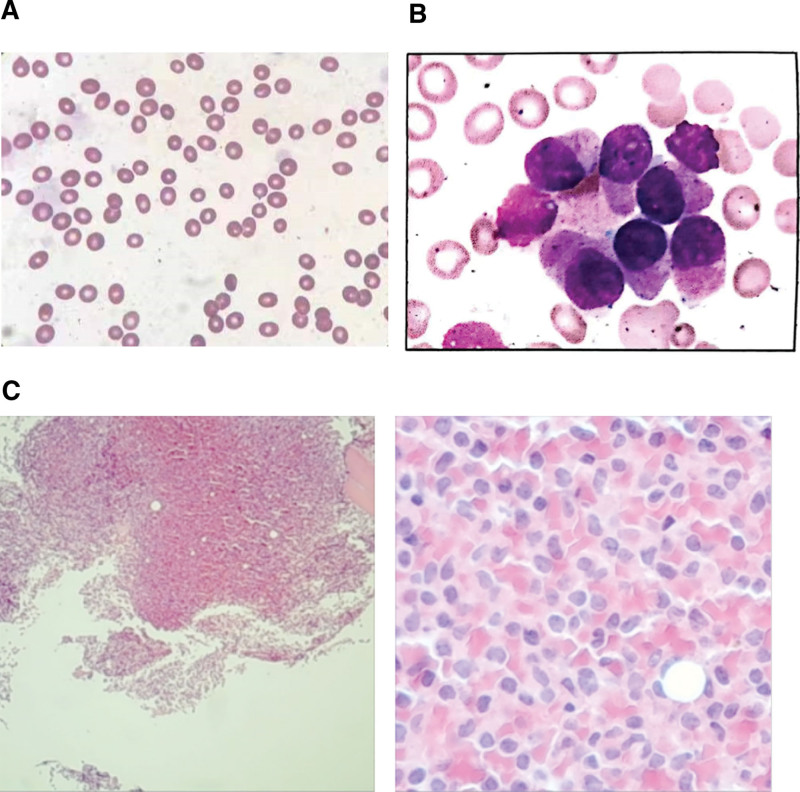
Morphologic features in peripheral blood, bone marrow aspirate and bone marrow biopsy at the time of diagnosis with APL. (A) Morphologic review of the peripheral blood smear revealed that too few white blood cells to count, the red blood cells size varies slightly, and more platelets can be seen. (B) Bone marrow aspirate at the time of diagnosis with APL showed a marked hypercellularity with 2.5% myeloblasts and 87% promyelocytes, the occurrence of faggot cells, 3.5% lymphocytes, erythroid hyperplasia was suppressed, and 3 megakaryocytes were seen in the whole film. (C) Bone marrow biopsy at the time of diagnosis with APL revealed hypercellular marrow (>90%), diffuse blasts with fine chromatin, round or irregular nuclei, abundant cytoplasm, scattered erythrocytes, and a lack of megakaryocytes. Mesh in protein staining was consistent with grade 1 bone marrow fibrosis. APL = acute promyelocytic leukemia.

**Figure 5.**
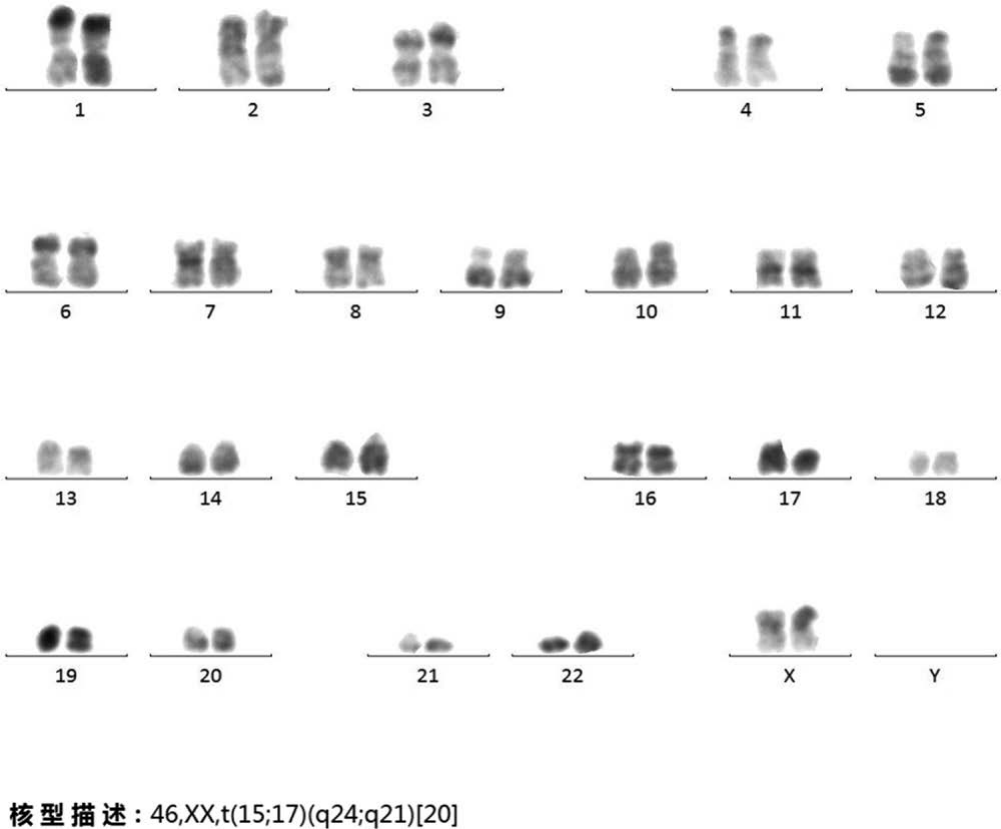
Cytogenetic analysis of the fresh bone marrow aspirate revealed 46, XX, t(15; 17)(q24; q21)[20].

**Figure 6.**
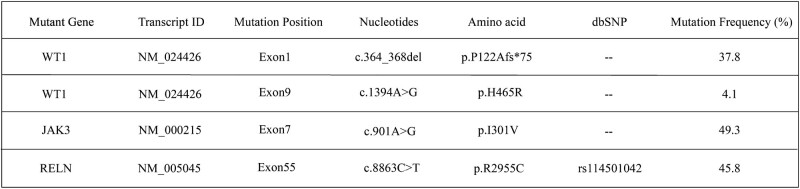
Genetic tests revealed that JAK2/V617F was negative, PML/RARαV variant (23.85% mutation load), WT1-Exon1 (37.8%), WT1-Exon9 (4.1%), JAK3-Exon7 (49.3%), and RELN-Exon55 (45.8%). dbSNP = The Single Nucleotide Polymorphism Database, ID =Identity document.

In June 2021, the patient complained of fatigue and poor sleep quality for 2 months. The CBC findings of the peripheral blood in July 2019 were as follows: WBC 1.87 × 10^9^/L, neutrophils 1.02 × 10^9^/L, Hb 137 g/L, and PLT 290 × 10^9^/L. The patient continued to take oral hydroxyurea (2 tablets/d). The repeated CBC in August 2021 revealed WBC 1.55 × 10^9^/L, neutrophils 0.31 × 10^9^/L, Hb 104 g/L, and PLT 25 × 10^9^/L. Morphologic review of the peripheral blood smear revealed that too few WBCs to count, the red blood cells size varies slightly, and more PLTs can be seen (Fig. 4A). BM aspirate was examined in an effort to establish the diagnosis, showing a marked hypercellularity with 2.5% myeloblasts and 87% promyelocytes, the occurrence of faggot cells, 3.5% lymphocytes, erythroid hyperplasia was suppressed, and 3 megakaryocytes were seen in the whole film (Fig. 4B). BM biopsy revealed hypercellular marrow (>90%), diffuse blasts with fine chromatin, round or irregular nuclei, abundant cytoplasm, scattered erythrocytes, and a lack of megakaryocytes. Mesh in protein staining was consistent with grade 1 BM fibrosis (Fig. 4C). Flow cytometry analysis showed that 93.36% of the cells showed high expression of CD117, CD33, CD13, CD123, MPO, and CD9, with dim expression of CD64 and CD38 and lack of expression of CD34 and CD7, confirming the diagnosis of APL. Cytogenetic analysis of the fresh BM aspirate revealed 46, XX, t(15; 17)(q24; q21)[20] (Fig. 5). Genetic tests revealed that JAK2/V617F was negative, PML/RARαV variant (23.85% mutation load), WT1-Exon1 (37.8%), WT1-Exon9 (4.1%), JAK3-Exon7 (49.3%), and RELN-Exon55 (45.8%) (Fig. 6).

### 1.2. Management and treatment

Based on the patient’s medical history, peripheral blood cell morphology, BM pathology, and genetic testing, we considered the following diagnosis: myelodysplastic syndrome, which represents a heterogeneous group of myeloid disorders characterized by severe cytopenia and dysplasia in 1 or more myeloid lineages. This patient experienced cytopenia, but the other criteria were not met. Infectious diseases: the patient had no fever, cough, local swelling, pain, or other symptoms of infection; all infection indexes were also negative, so we excluded infectious diseases. Consequently, she was diagnosed with APL transformed from PV (medium risk) based on World Health Organization Classification of Tumors. Pathology and Genetic of Tumors of Hematopoietic and Lymphoid Tissue.

She was initially treated with combined all-trans retinoic acid (ATRA) and arsenic trioxide. She received additional treatment with oral hydroxyurea 1.0 tid when the leukocytes were more than 4 × 10^9^/L and daunorubicin at more than 10 × 10^9^/L to lower the leukocytes. She had a prolonged corrected QT interval on electrocardiography during induction therapy. Arsenic trioxide therapy was subsequently adjusted to compound Huangdai tablets, and no prolongation of the corrected QT interval was observed.

**Figure 7.**
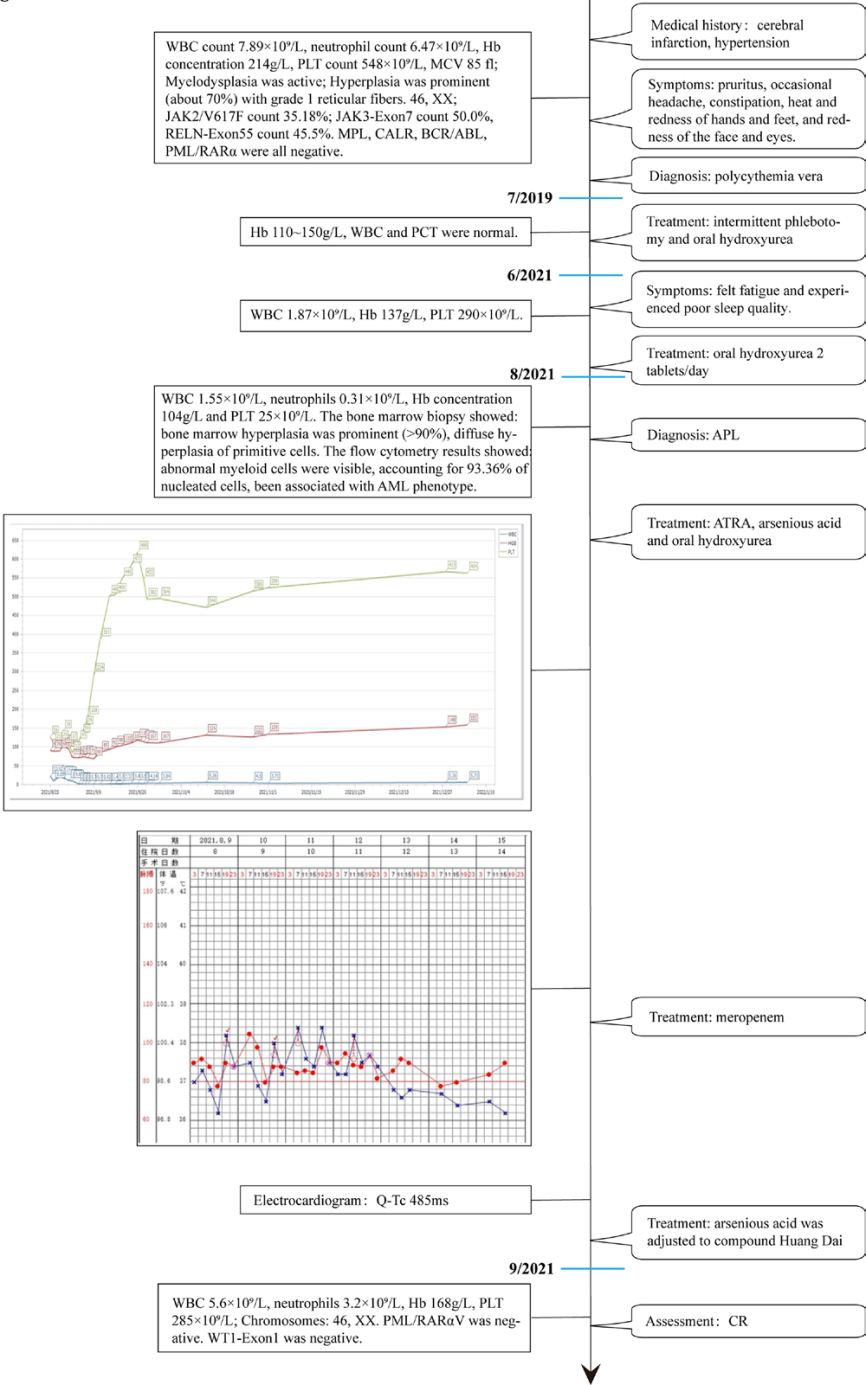
Timeline of interventions and outcomes. AML = acute myelocytic leukemia, APL = acute promyelocytic leukemia, ATRA = all-trans-retinoic acid, CR = complete remission, Hb = hemoglobin, MCV = mean corpuscular volume, PLT = platelet, QTc = corrected QT, WBC = white blood cell.

After 28 days of induction therapy, the CBC findings revealed a WBC 5.6 × 10^9^/L, neutrophils 3.2 × 10^9^/L, Hb level of 168 g/L, and PLT 285 × 10^9^/L. BM aspirate showed active BM hyperplasia (90%), trilineage hematopoietic hyperplasia, and no remarkable hyperplasia in blasts. BM fibrosis progressed to grade 2. Cytogenetic analysis revealed a 46, XX karyotype. Genetic tests revealed PML/RARαV and WT1-Exon1 were negative. She achieved complete remission (CR) (Fig. 7) and was compliant, and the treatment was well tolerated throughout. She had an episode of neutropenia with fever; however, her temperature improved with anti-infective therapy, and no serious infectious events occurred. The patient provided informed consent for publication of this case.

## 2. Discussion

Transformation of the PV into the APL is very rare. To our knowledge, this is the third reported case of transformation of a patient from PV to APL after receiving intermittent phlebotomy and oral hydroxyurea. In this case, the patient was diagnosed with PV with the wild-type JAK2/V617F variant (49.1% mutation load), but MPL, CALR, BCR/ABL, and PML/RARα were all negative. However, when she was diagnosed with APL, genetic tests revealed JAK2/V617F as negative, PML/RARαV variant (23.85% mutation load), WT1-Exon1 (37.8%), WT1-Exon9 (4.1%), JAK3-Exon7 (49.3%), and RELN-Exon55 (45.8%). In conclusion, after transformation to APL, the JAK2/V617F mutation disappeared, and the PML/RARα and WT1 genes became positive. The patient was considered to have clonal evolution at the molecular level.

Transformation to APL in patients with PV usually occurs several years after PV diagnosis. To date, only 3 cases of transformation to APL have been reported. There were 2 men and 1 woman in the 3 cases 59, 45, and 51 years old, respectively. These 3 patients developed APL within 2, 1.25, and 2 years after the diagnosis of PV, respectively. They underwent phlebotomy alone and intermittent phlebotomy combined with hydroxyurea as the initial therapy (Table 1).^[8,9]^

**Table 1:** Patient characteristics.

Author (references)	Patient sex and age, y	Before transformation					After transformation				
		Diagnosis	Karyotype	Mutation status	Duration from PV to APL, y	Treatment	Karyotype	Mutation status	Treatment	Outcome	Follow-up time from transformation, y
Mollee et al^[8]^ (1999)	Male, 59	PV	NA	NA	2.00	Phlebotomy	46, XY, t(15;17)	BCR-1/2	Chemotherapy	CR	2.00
Kajiguchi et al^[9]^ (2000)	Male, 45	PV	46, XY	NA	1.25	Phlebotomy	46, XY, t(15;17)	PML/RARα	ATRA, chemotherapy	CR	0.92
This case	Female, 51	PV	46, XX	JAK2/V617F	2.00	Phlebotomy, hydroxyurea	46, XX, t(15;17)	PML/RARα, WTI-Exon1, WTI-Exon9, JAK3-Exon7, RELN-Exon55	ATRA, chemotherapy, arsenious acid	CR	NA

In comparison, the rate of patients with ET transforming into APL was higher than that of patients with PV. Five patients (4 men and 1 woman) experienced transformation of ET to APL, with a median age of 51 years (range, 31–57 years). One patient had an abnormal karyotype (46, XY, -21, +dic[21]) at the initial diagnosis, whereas the others had a normal karyotype. The duration of transformation from ET to APL was 65 months (range, 19–111 months). Two of the 5 patients had an abnormal karyotype (t[15;17]) after transformation, and the others had normal karyotypes. Chemotherapy was mostly used after transformation, and 1 patient received a combination of ATRA and the other received allogeneic peripheral blood stem cell transplantation. Two of the 5 patients achieved CR, and 3 died from complications (n = 2) or after relapse (n = 1). The median of overall survival time after transformation was 2.21 years (range, 0.67–3.50 years). The detailed patient characteristics are shown in Table 2. Thus, patients with ET-transformed APL appeared to have shorter survival times than those with primary APL. However, the number of cases was too small to draw any conclusions in the present study, so it should be further identified.^[10–13]^

**Table 2:** Patient characteristics.

Author (references)	Patient sex and age, y	Before transformation					After transformation			
		Diagnosis	Karyotype	Duration from ET to APL, y	Treatment	Karyotype	Mutation status	Treatment	Outcome	Follow-up time from transformation, y
Ratti et al^[10]^ (1984)	Male, 36	ET	46, XY	3.75	Uramustine	46, XY	Not done	Chemotherapy	CR	NA
Sessarego et al^[11]^ (1989)	Male, 31	ET	46, XY	5.17	Busulfan	46, XY	Not done	NA	Dead	0.67
Lofvenberg et al^[12]^ (1990)	Male, 57	ET	46, XY, –21, +dic (21)	1.58	Hydroxyurea	NA	Not done	Chemotherapy	Dead	2.58
Mollee et al^[8]^ (1999)	Female, 51	ET	46, XX	4.00	Hydroxyurea, warfarin	46, XX, t(15;17)	BCR-1/2	ATRA, chemotherapy	CR	3.50
Sato et al^[13]^ (2002)	Male, 54	ET	46, XY	9.25	None	46, XY, add(8) t(15;17)	BCR-3	Chemotherapy, allo-PBSCT	Dead by relapse	1.83

Transformation of myelofibrosis into APL is even rarer. To date, only 1 case has been reported. A 66-year-old woman with a normal karyotype was diagnosed with primary myelofibrosis. Transformation to APL occurred 80 months after hydroxyurea treatment, and she achieved CR and long-term survival with chemotherapy combined with ATRA treatment.^[14]^

To date, there have been few reports regarding the transformation of MPN into APL. Patients were mostly treated with chemotherapy in the past; as ATRA and arsenic trioxide became available, more patients received standard treatment for APL. However, the number of cases was too small to compare the efficacy of transformation into APL and primary APL.

In this case, the patient received hydroxyurea intermittently for approximately 2 years at the time of PV diagnosis. Hydroxyurea has the potential to cause malformations and induce second tumors.^[15,16]^ Therefore, the possibility of hydroxyurea-induced APL development cannot be excluded at this time. However, most studies have reported that hydroxyurea induces solid tumors. There are only 3 reported cases of MPN transformation to AML after receiving hydroxyurea,^[13]^ the median time of transformation to APL was 4 years, and 66.7% of patients were ≥ 2 years. Therefore, it is unlikely that the transformation in this case was triggered by hydroxyurea.

This study has some limitations. In this case, there was still not enough objective evidence of clonal evolution because the patient did not regularly monitor the BM for molecular biology due to personal financial reasons. This case suggests that bone aspiration and biopsy every 3 to 6 months during PV treatment to perform a comprehensive assessment and monitor the disease status, which can detect disease progression and intervene early when it transforms into APL. This was also recommended by the National Comprehensive Cancer Network guidelines.

## Acknowledgments

We are very grateful to all the participants of the study.

## Author contributions

Conceptualization: Xiao-Dong Mo. Data curation: Wen-Wen Li, Xiu-Fang Sui. Writing—original draft: Wen-Wen Li, Shuang Fan, Hong Xu. Writing—review & editing: Shuang Fan, Cheng-Lei Wang, Fei-Ying Wang.
